# Athlete Self-Report Measure Use and Associated Psychological Alterations

**DOI:** 10.3390/sports5030054

**Published:** 2017-07-26

**Authors:** Anna E. Saw, Luana C. Main, Sam Robertson, Paul B. Gastin

**Affiliations:** 1 Centre for Sport Research (CSR), Deakin University, Geelong 3220, Australia; paul.gastin@deakin.edu.au; 2 Institute for Physical Activity and Nutrition (IPAN), Deakin University, Geelong 3220, Australia; luana.main@deakin.edu.au; 3 Institute of Sport, Exercise and Active Living (ISEAL), Victoria University, Melbourne 8001, Australia; sam.robertson@vu.edu.au

**Keywords:** confidence, motivation, satisfaction, self-awareness, self-regulation, monitoring

## Abstract

The experience of athletes and practitioners has led to the suggestion that use of an athlete self-report measure (ASRM) may increase an athlete’s self-awareness, satisfaction, motivation, and confidence. This study sought to provide empirical evidence for this assertion by evaluating psychological alterations associated with ASRM use across a diverse athlete population. Athletes (*n* = 335) had access to an ASRM for 16 weeks and completed an online survey at baseline, and weeks 4, 8, and 16. Generalized estimating equations were used to evaluate the associations between ASRM compliance and outcome measures. Compared to baseline, confidence and extrinsic motivation were most likely increased at weeks 4, 8, and 16. Satisfaction and intrinsic motivation were most likely decreased at week 4, but no different to baseline values at weeks 8 and 16. Novice athletes and those who were instructed to use an ASRM (rather than using one autonomously) were less responsive to ASRM use. This study provides preliminary evidence for ASRM to prompt initial dissatisfaction and decreased intrinsic motivation which, along with increased confidence and extrinsic motivation, may provide the necessary stimulus to improve performance-related behaviors. Novice and less autonomous athletes may benefit from support to develop motivation, knowledge, and skills to use the information gleaned from an ASRM effectively.

## 1. Introduction

Many athletes engage in the practice of routinely recording their training and performance-related behaviors (e.g., nutrition, recovery, mental preparation), along with their perceived wellbeing (e.g., mood, fatigue, soreness). The instruments used to record such information, be it paper or electronically, can collectively be termed athlete self-report measures (ASRM). The act of completing an ASRM has anecdotally been suggested to increase athlete self-awareness and ownership of their preparation which may, in turn, lead to better training and performance-related behaviors [1,2,3,4]. This is a promising role of ASRM which warrants empirical investigation.

The efficacy of self-monitoring for positive behavior change is well supported, theoretically. From a social cognitive perspective [5], self-monitoring is an essential component of self-regulated behavior change [6]. The self-regulation theory [7] presents a cyclical process whereby self-monitoring of behaviors informs future behavioral adjustments. An alternate, or possibly complementary, theory is that of self-determination [8], whereby self-monitoring influences motivation and, in turn, behaviors. Research on self-monitoring in athletes is in its infancy; hence, the present study adopts an exploratory approach rather than adopting a particular theoretical model. The outcome measures of interest (self-awareness, satisfaction, motivation, and confidence) reflect psychological indices commonly raised anecdotally, and which have also been theorized to make positive behavior change more likely [9].

Conceptually, self-awareness forms part of the iterative process between the components of self-monitoring: observation, measurement, and recording [10]. It is postulated that the intention to use an ASRM may prompt self-awareness in order for the athlete to consciously observe, measure, and record their behaviors. Similarly, the recorded information may serve to increase self-awareness. This is illustrated by the following quote from an elite athlete: *“an ASRM lets you reflect on yourself at that moment. You actually have to think about what you’re doing, and all your plans, and how you’re actually treating your body”* [11] (p. 208). Increased self-awareness is also a recurring theme reported by practitioners and researchers alike who implement ASRM with their athletes [1,2,3,4].

In addition to increased self-awareness, use of an ASRM *“makes [athletes] identify things that they do well or don’t do well”* [11] (p. 208). Evaluation of behaviors and wellbeing against goals and personal standards will determine the degree of satisfaction an athlete has for their current preparation [5]. If the discrepancy between actual and goal behaviors is reduced, satisfaction may be increased. For instance, one athlete reported that *“...when I see how much I have done, I commend myself for my efforts. This is important as it helps me stay positive.”* [12] (unpublished data). However, failure to progress towards a goal may equally prompt dissatisfaction [5].

It is possible that both satisfaction and dissatisfaction with behaviors and wellbeing may provide a motivational stimulus for the athlete. Athletes have reported that they used their strengths and weaknesses identified through an ASRM *“for future inspiration”* and *“to motivate myself to train harder than in the past, and continue to improve.”* [12] (unpublished data). In this instance, the motivation gained from engaging in athletic preparation and self-monitoring may strengthen the motivation of the initial athletic goal [13]. Alternatively, dissatisfaction may reduce motivation for athletic preparation and, therefore, detract from the initial athletic goal, potentially resulting in the revising down of the goal.

The motivation to improve athletic preparation may affect, and be affected by, the confidence an athlete has in their abilities to do so. Confidence is determined by various sources of information which an athlete uses to judge their ability [14]. An ASRM may be one such source, directing an athlete’s attention to controllable factors, which is associated with increased confidence [14]. For instance, an athlete who was highly driven towards their Olympic goal was described by their coach as using an ASRM to ensure they ‘ticked all the boxes’:

“One of the guys is quite keen to be able to say to himself when he sits on the start line: ‘Have I done everything I possibly could?’. And if he says ‘yes’ every day to that point, then he’s confident to race”.

*[11] (p. 212)*

Only one study has evaluated the effect of ASRM use on athlete behaviors and related psychological indices in an ecologically-valid setting. Intercollegiate swimmers (*n* = 26) who completed either a log on salient training behaviors 5–6 times per week, or a daily log on peripheral aspects of training (e.g., sleep, nutrition, stress, soreness) for 26 days reported a heightened intention to assess and improve their training behaviors, and greater confidence in their ability to do so [15]. As such, there is a need to replicate this promising finding, to determine whether the increased confidence is maintained, further increased, or diminished with longer-term use of an ASRM, and whether this finding occurs in other athlete populations.

Taken together, there is qualitative and theoretical support for an ASRM to improve athlete self-awareness, satisfaction, motivation, and confidence. There is also limited quantitative evidence for ASRM use to increase confidence over the short term in competitive athletes. However, qualitative findings suggest this may occur for some athletes and not others. Individual factors, such as expertise, intrinsic motivation for their sport, and motivational disposition towards an ASRM need to be considered.

Antecedents for self-monitoring in athletes likely include an individual’s knowledge of athletic preparation, skills for observation, measurement, and problem solving, goal-setting, and social support [10]. Each of these antecedents have been shown to be superior in expert athletes [16,17,18,19]. Furthermore, experts may place a higher value and apply greater effort towards their athletic preparation in order to perform, and also to satisfy their sense of personal adequacy [5]. Therefore, expert athletes may be better placed to effectively use an ASRM for self-monitoring. However, this is yet to be established.

Whilst certain characteristics of knowledge, skills, and perceived importance of athletic preparation may by typified by expertise, this is not absolute. It may also be relevant to consider an athlete’s motivational disposition for their sport, and also for using an ASRM. An athlete who is more intrinsically motivated to participate in their sport should have a more process-orientated approach [20]. It is postulated that a process-orientated approach to athletic preparation is favorable for ASRM use. Therefore, it should be investigated whether athletes who are more intrinsically motivated to participate in their sport benefit more from ASRM use.

Motivation to use an ASRM may be of an autonomous or controlled origin. An athlete who actively chooses to use an ASRM on their own accord may be deemed to be autonomously motivated. Athletes who are autonomously motivated may be better positioned to persist with ASRM use and, hence, benefit over the longer-term [21,22]. However, it is common for athletes to be instructed to use an ASRM by their coach or sports program. Often in this situation, the coach, and possibly other practitioners, have access to the athlete’s ASRM data, and may use the information to assist their decision-making. Athletes using an ASRM under instruction may possess controlled motivation, characterized by external regulation along with introjected regulation to gain approval or avoid punishment [8]. Therefore, motivational disposition towards an ASRM should also be considered. As such, the aim of the present study was to evaluate whether athletes of different expertise and motivation to self-monitor may benefit from ASRM use through increased self-awareness, satisfaction, motivation, and confidence.

## 2. Materials and Methods

### 2.1. Participants

Of the 396 athletes who completed the baseline measures, 335 (85%) returned to complete one or more follow-up surveys and, hence, are reflected in the results. There were no differences in characteristics nor baseline psychometric indices between athletes who completed repeat surveys and those who did not. Athletes (32.9 ± 14.3 years, 61% male) reported their participation level as recreational (19%) or competing at a club or university (32%), regional (24%), national (19%), or international (6%) level. Athletes were either instructed to use an ASRM by their sports organization (termed ‘controlled’, 18%), or sought to use an ASRM themselves (termed ‘autonomous’, 82%).

### 2.2. Athlete Self-Report Measure

Specialized online software provided athletes with a platform to record their training and wellbeing, along with other training-related information (e.g., goals, program, performance, nutrition, body weight, injury, illness) (Metrifit, Health and Sport Technologies Ltd., Greenore, Ireland). Wellbeing questions (mood state, sleep quality, sleep duration, energy levels, muscle readiness, appetite) were required to be completed daily, with compliance supported by optional prompts and reports. Athletes received instruction on how to use the program and could utilize the online support feature.

### 2.3. Survey Instruments

#### 2.3.1. Self-Awareness

Self-awareness was evaluated using three factors of the Kentucky Inventory of Mindfulness Skills (KIMS) (observe, describe, act with awareness, 30 items) [23]. The fourth factor, accept without judgment, was excluded as the evaluation of behaviors and wellbeing against goals and personal standards has been described as an integral component in how ASRM use may affect psychological indices and behavior change. Statements were rated on a five-point Likert scale from 1 (never or very rarely true) to 5 (very often or always true). The Cronbach’s alpha internal reliability coefficient for items contributing to the measure of self-awareness in the present study was 0.86.

#### 2.3.2. Athlete Satisfaction

Satisfaction was evaluated using two pertinent subscales of the Athlete Satisfaction Questionnaire (ASQ) (individual performance, personal dedication, seven items) [24]. Athletes rated their satisfaction on a seven-point Likert scale from 1 (not at all satisfied) to 7 (extremely satisfied). The Cronbach’s alpha for items contributing to the measure of satisfaction in the present study was 0.96.

#### 2.3.3. Sport Motivation

Intrinsic and extrinsic motivation and amotivation were evaluated using the Sport Motivation Scale (SMS) (28 items) [20]. In response to the question ‘*Why do you practice your sport?’* athletes respond to statements on a seven-point Likert scale from 1 (does not correspond at all) to 7 (corresponds exactly). For the present study, Cronbach’s alpha for intrinsic, extrinsic, and amotivation were 0.90, 0.85, and 0.82 respectively.

#### 2.3.4. State Sport-Confidence

Confidence was evaluated using the State Sport-Confidence Inventory (SSCI) (13 items) [25]. Athletes are asked to reflect on how confident they are feeling right now about an upcoming competition or event, relative to the most self-confident athlete they know, and respond on a nine-point Likert scale from 1 (low) to 9 (high). The Cronbach’s alpha in the present study was 0.96.

#### 2.3.5. Additional Questions

The baseline questionnaire included demographics and previous and concurrent use of other ASRM and training records. Additional factors which may influence compliance and psychological state surveyed at each time point included current training phase (volume, intensity, time to competition), and time lost to injury or illness in the past four weeks.

### 2.4 Procedure

The study procedure was approved by the university human ethics advisory group, and conducted independently of the ASRM provider and sports organizations. This allowed ASRM use to evolve organically. Athletes were invited to complete the baseline electronic survey (QuestionPro Inc., San Francisco, CA, USA) on their own device. Athletes who completed the baseline survey were contacted via email at weeks 4, 8, and 16, with up to three reminders sent as necessary. Athletes were allowed one week within which to complete the required survey at each time point. Athletes were informed that the study was investigating how using an ASRM may help them, however, the specific research question was not disclosed to avoid introducing bias.

### 2.5 Analysis

Compliance was calculated as a percentage of daily ASRM wellbeing entries over the preceding 28 days. Quasi-subgroups for expertise were inferred from reported participation levels in-line with previous classifications [18,19]. Athletes competing at a national or international level were classified as ‘experts’, those competing at a club, university or regional level were classified as ‘non-experts’, and recreational athletes were classified as ‘novices’.

Scores from questionnaires were converted to percentages to aid interpretation and comparison. Outcome measures satisfied the assumption of normality, with the exception of amotivation which was positively skewed. Amotivation data was log transformed for analyses, and back transformed for reporting in results.

Descriptive statistics were calculated for each outcome measure at baseline. Independent samples *t*-tests compared subsets of athletes grouped by expertise and ASRM motivation. Generalized estimating equations with an exchangeable correlation structure and identity link function were used to evaluate the association between compliance and outcome measures at weeks 4, 8, and 16. Previous use of an ASRM, concurrent use of a different ASRM or training record, training load, time to competition, and injury or illness were controlled for in the models. All analyses were performed in SPSS (Version 24.0. IBM Corp., Armonk, NY, USA), and statistical significance was set at *p* < 0.05 for all models. The practical importance of findings were determined using magnitude based inferences [26].

## 3. Results

Participant characteristics for all athletes, and athletes grouped by expertise and motivation to use an ASRM are summarized in Table 1. Some differences in baseline psychological measures were noted between athletes of different expertise, and between athletes with autonomous and controlled motivations to use an ASRM (Table 2). Experts reported a mean satisfaction which was approximately 4% higher than novice athletes (*p* = 0.017), and non-experts reported satisfaction approximately 6% higher than novice athletes (*p* < 0.001). Experts reported a mean confidence which was approximately 11% higher than novice athletes (*p* < 0.001) and 6% higher than non-experts (*p* = 0.003). Non-experts reported a mean confidence which was approximately 6% higher than novice athletes (*p* = 0.010). Experts reported a mean intrinsic motivation which was approximately 4% higher than novice athletes (*p* = 0.015). Non-experts reported a mean extrinsic motivation which was approximately 3% higher than novices (*p* = 0.031). Autonomously motivated athletes reported a mean self-awareness which was approximately 4% higher than athletes with controlled motivation (*p* < 0.001).

Compliance and training load were consistent across each time point, with a mean compliance of 21.9 ± 37.5% (range 0–100%), and training load of 11.1 ± 4.1 arbitrary units. Throughout the study, the percentage of athletes using another ASRM ranged from 0.8–1.8%, and those who recorded their training elsewhere ranged from 52.4–57.0%. At week 4, 9.7% of athletes had experienced an injury or illness which prevented them from training for any period during the previous four weeks, and a further 26.1% experienced an injury or illness which required them to modify training. At week 8 these incidences were 13.4% and 23.6%, and at week 16, 7.2% and 26.4%, respectively. Each of these observations are controlled for in the following results.

Across all athletes, ASRM use was most likely associated with a trivial decrease in satisfaction (*p* < 0.001) and intrinsic motivation (*p* = 0.053) at week 4 (Table 1). ASRM use was most likely associated with a trivial increase in confidence at week 4 (*p* = 0.006), 8 (*p* = 0.010), and 16 (*p* = 0.029). ASRM use was most likely associated with a trivial increase in extrinsic motivation at week 4 (*p* < 0.001), 8 (*p* = 0.004), and 16 (*p* < 0.001). ASRM use was likely-very likely associated with an increase in amotivation at week 4 (*p* = 0.031), 8 (*p* = 0.092), and 16 (*p* = 0.013). There was no association between ASRM use and changes in self-awareness at any time point.

When expertise was included as a main effect in the model (Table 2, part a), non-experts and experts who were more compliant with ASRM use reported a most likely trivial decrease in satisfaction at week 4 (*p* < 0.001, *p* = 0.001 respectively). Experts had a more marked increase in both confidence (week 4 *p* = 0.002, week 8 *p* = 0.002, week 16 *p* = 0.018) and extrinsic motivation (weeks 4, 8, and 6 *p* < 0.001). Non-experts also reported a most likely trivial increase in extrinsic motivation at week 16 (*p* = 0.023). Novice athletes reported a most likely trivial decrease in self-awareness (*p* = 0.002) and intrinsic motivation (*p* = 0.016) at week 16.

When motivational disposition for using an ASRM was included as a main effect in the model (Table 2, part b), autonomous athletes who were more compliant with ASRM use reported a most likely trivial decrease in satisfaction at week 4 (*p* < 0.001). Autonomous athletes had a more marked increase in confidence at week 4 (*p* = 0.004), 8 (*p* < 0.001), and 16 (*p* = 0.003). Autonomous athletes also reported a most likely trivial increase in intrinsic motivation at week 16 (*p* = 0.036).

## 4. Discussion

The experience of athletes and practitioners has led to the suggestion that use of an ASRM may increase an athlete’s self-awareness, satisfaction, motivation, and confidence [1,2,3,4]. The present study sought to provide preliminary empirical evidence for this assertion by evaluating psychological alterations associated with ASRM use across a diverse athlete population. Preliminary evidence supports a trivial increase in confidence and extrinsic motivation across the first 16 weeks of ASRM use. Contrary to previous assertions, ASRM use was most likely associated with a trivial decrease in satisfaction and intrinsic motivation at week 4, which returned to baseline levels at weeks 8 and 16. ASRM use was also likely-very likely associated with an increase in amotivation across the 16 weeks. It should be noted that although psychological alterations were found to be trivial, modelled changes are for every 1% increase in ASRM compliance. For example, confidence (B ~ 0.10) may increase by 1% with a 10% increase in compliance, and by 5% with a 50% increase in compliance. In practice, such changes are substantial, particularly at the expert level.

The association between ASRM use and increased confidence is an encouraging finding. Confidence is a positive predictor of behavior change [27,28], thus supporting the potential for ASRM to lead to improved training and performance-related behaviors. The observed increase in confidence with ASRM use partially agrees with the findings of Young, Medic, and Starkes [15], who observed increased confidence in less than four weeks in non-expert swimmers with a controlled motivational disposition towards their ASRM use. In the present study, non-experts had a likely-very likely increase in confidence, whilst findings were unclear for controlled athletes. However, the possibility that other sources of confidence (e.g., coach, social support, vicarious experience, and environmental and situational factors [29]) may have confounded the results of either study should also be considered.

To exploit the possible influence of ASRM use on confidence and behavior change, ASRM questions should focus on success and behaviors which are relatively easy to change [5]. Monitored behaviors should also be specific to an athlete’s sport, goals, and expertise to increase interest [5,16,18,30]. Self-monitoring should be performed regularly, in close temporal proximity to the behaviors being monitored, and in a supportive environment [5,31].

Extrinsic motivation increased alongside confidence, supporting a possible interplay between psychological indices as per the self-regulation model [7]. For instance, an increase in confidence for an upcoming competition or event may strengthen the extrinsic performance goal. An alternative explanation is that more extrinsically-motivated athletes may have been more compliant with ASRM use. This may explain the seemingly contradictory concurrent increase in amotivation.

A possible explanation for the decrease in satisfaction at week 4 is that use of an ASRM caused athletes to become more aware that their athletic practices were incongruent with their personal goals. A perceived lack of competence may also explain the accompanying decrease in intrinsic motivation [13]. Whilst these psychological responses are negative, they may provide the necessary stimulus to prompt improved behaviors in the future. Therefore, athletes and practitioners should be aware that dissatisfaction and reduced intrinsic motivation may be an initial consequence of ASRM use, however, this response is temporary and may precede positive outcomes.

Use of an ASRM seemed less effective for novice athletes. A decrease in intrinsic motivation reported by novice athletes at week 16 may reflect a delayed response to that typically observed at week 4 for other athletes. This may reflect the time taken to develop the necessary knowledge and skills to effectively self-monitor. However, a corresponding decrease in self-awareness may also simply reflect changing interest in sport over time amongst novice athletes. The goal of novice athletes may not necessarily have been to improve athletic behaviors and performance, hence, reducing the efficacy of ASRM use [13]. However, this generalization should not preclude novice athletes from using an ASRM.

Use of an ASRM seemed less effective for athletes with a controlled motivational disposition. The apparent impediment of motivational disposition may be overcome with time, as previous research has shown athletes to develop a positive attitude towards an ASRM following a season of controlled use [32]. However, in the present study over 16 weeks, there was no apparent convergence in findings between the groups which may have suggested increased autonomous motivation amongst controlled users. Sports organizations must, therefore, be prepared to persist with efforts to support ASRM use throughout the season in order for athletes to experience potential psychological benefits from ASRM use.

Practitioners may play an important role in supporting novice athletes and those in a controlled ASRM setting to engage with and benefit from ASRM use. One such role is engaging in ‘co-regulation’ whereby the practitioner assists an athlete to monitor and self-regulate their behaviors until they are able to do so independently [33]. For instance, practitioners may assist an athlete to develop skills to effectively self-monitor their athletic preparation by encouraging an athlete to be accountable for their behaviors, make effective judgments, and attribute errors to controllable factors. Practitioners may also contribute to the athlete’s sense of satisfaction by providing praise, positive reinforcement, and constructive feedback. Motivation and confidence may be aided by guiding the athlete to set goals, plan, select appropriate strategies, and to anticipate and prepare for challenges. Where ASRM data is being concurrently used by practitioners, efforts should also be directed at creating a supportive environment such that the athlete does not feel the need to shade their responses in order to manage their self-presentation [5,31,34].

A limitation of the present study is the inability to control for the multitude of factors which influence athletes’ psychological state, including the support offered by practitioners. The present study findings are also limited to the ASRM utilized or similar multidimensional, online measures. Another methodological limitation is the unbalanced athlete numbers across the different subgroups, however, this is handled by the analytical approach. Self-reported outcome measures are informative, yet susceptible to bias [35,36]. To minimize the risk of bias, the present study followed the recommendations of Podsakoff et al. [36] to provide athletes with clear instruction and assure anonymity. Furthermore, the analytic approach evaluated intra-individual changes over time, thus any systematic bias may have been inconsequential.

## 5. Conclusions 

Athletes who use an ASRM to self-monitor their behaviors and perceived wellbeing may experience changes in various psychological indices. In the present study, athletes experienced a temporary decrease in satisfaction and intrinsic motivation following four weeks of ASRM use. This disruption may provide a useful prompt to improve behaviors. Athletes who persisted with ASRM use experienced increased confidence and extrinsic motivation. Increased confidence is a positive predictor of behavior change, supporting the potential for ASRM use to lead to improved training and performance-related behaviors. Novice athletes and those who were instructed to use an ASRM were less responsive to ASRM use. These athletes may still benefit from ASRM use provided they have, or develop, the motivation, knowledge, and skills to use the information gleaned from an ASRM effectively. Practitioners may facilitate this process by supporting the athlete to self-monitor and self-regulate their behaviors. These preliminary findings support a potentially valuable role of athlete self-report measures in psychological wellbeing and self-regulation which warrants further investigation.

## Figures and Tables

**Table 1:** Participant characteristics presented as percentages for all athletes, and athletes grouped according to **a**) expertise, and **b**) motivation to use an ASRM.

Athlete Group		Expertise	ASRM Motivation	Gender	Sport	Previous ASRM Experience
Subgroup		Novice	Autonomous	Male	Individual	Other ASRM
		Non-expert	Controlled	Female	Team	Training record
		Expert				None
**All** (*n* = 335)		19	82	61	68	8
		56	18	39	32	58
		25				34
**a)**	**Expertise**					
	Novice (*n* = 64)	-	100	62	91	13
			0	38	9	59
						28
	Non-expert (*n* = 188)	-	77	66	64	6
			23	34	36	54
						40
	Expert (*n* = 83)	-	81	46	60	9
			19	54	40	63
						28
**b)**	**ASRM motivation**					
	Autonomous (*n* = 275)	23	-	58	82	10
		52		42	18	62
		24				28
	Controlled (*n* = 60)	0	-	73	3	2
		73		27	97	36
		27				62

**Table 2:** Changes in psychological indices (self-awareness, satisfaction, confidence, motivation) associated with 16 weeks of ASRM use for all athletes (*n* = 335), and athletes grouped according to **a**) expertise (novice *n* = 64, non-expert *n* = 188, expert *n* = 83), and **b**) motivation to use an ASRM (autonomous *n* = 275, controlled *n* = 60).

Psychological Index		Baseline	Week 4	Week 8	Week 16	Inference
Athlete Subgroup		Mean (95% CI) *n* = 335	B (95% CI) *n* = 165	B (95% CI) *n* = 125	B (95% CI) *n* = 126	Weeks 4-16 collectively
**Self-awareness** (All athletes)		62.3 (61.5–63.1)	0.00 (−0.03–0.03)	−0.02 (−0.05–0.02)	−0.02 (−0.07–0.03)	Unclear-very likely trivial
**a)**	**Expertise**					
	Novice	62.3 (60.4–64.2)	-	−0.03 (−0.07–0.00)	−0.12 (−0.19–−0.05) *	Most likely trivial
	Non-expert	62.3 (61.2–63.5)	0.04 (−0.04–0.13)	−0.01 (−0.06–0.03)	−0.01 (−0.07–0.05)	Very likely trivial
	Expert	62.1 (60.4–63.8)	0.01 (−0.05–0.08)	0.01 (−0.08–0.10)	−0.01 (−0.09–0.07)	Very likely trivial
**b)**	**ASRM motivation**					
	Autonomous	62.9 (62.0–63.8)	0.00 (−0.03–0.02)	−0.01 (−0.03–0.02)	−0.03 (−0.07–0.00)	Very-most likely trivial
	Controlled	59.1 (57.1–61.0) §§	-	−0.19 (−1.10–0.71)	−0.17 (−1.14–0.79)	Unclear
**Satisfaction**		52.0 (50.8–53.2)	−0.08 (−0.12–−0.03) **	0.01 (−0.05–0.06)	−0.01 (−0.06–0.04)	Very-most likely trivial
**a)**	**Expertise**					
	Novice	47.4 (44.8–50.0)	-	0.03 (−0.10–0.15)	0.25 (−0.01–0.52)	Likely-very likely trivial
	Non-expert	53.7 (52.1–55.2) †	−0.17 (−0.26–−0.08) **	0.00 (−0.06–0.06)	−0.01 (−0.06–0.04)	Unclear-most likely trivial
	Expert	51.9 (49.6–54.2) †	−0.08 (−0.13–−0.03) *	0.01 (−0.09–0.11)	−0.04 (−0.13–0.04)	Unclear-most likely trivial
**b)**	**ASRM motivation**					
	Autonomous	51.7 (50.4–52.9)	−0.08 (−0.12–−0.04) **	0.01 (−0.04–0.07)	−0.02 (−0.07–0.02)	Very-most likely trivial
	Controlled	53.8 (51.0–56.6)	-	−0.01 (−0.81–0.78)	−0.02 (−0.83–0.79)	Unclear-very likely trivial
**Confidence**		65.6 (63.8–67.3)	0.07 (0.02–0.12) *	0.10 (0.02–0.17) *	0.09 (0.01–0.17) *	Very-most likely trivial
**a)**	**Expertise**					
	Novice	59.7 (55.8–63.6)	-	0.07 (−0.01–0.15)	−0.13 (−0.30–0.05)	Very likely trivial
	Non-expert	65.6 (63.3–67.9) †	0.25 (−0.12–0.62)	0.08 (−0.02–0.17)	0.09 (−0.02–0.19)	Likely-very likely trivial
	Expert	70.0 (66.6–73.4) †‡	0.11 (0.04–0.19) *	0.20 (0.07–0.32) *	0.13 (0.02–0.24) *	Very-most likely trivial
**b)**	**ASRM motivation**					
	Autonomous	65.2 (63.3–67.1)	0.18 (0.06–0.30) *	0.21 (0.10–0.32) **	0.20 (0.07–0.32) *	Most likely trivial
	Controlled	67.4 (63.1–71.7)	-	0.72 (−0.88–2.31)	0.74 (−0.90–2.37)	Unclear
**Intrinsic motivation**		52.4 (51.3–53.5)	−0.04 (−0.08–0.00)	0.02 (−0.03–0.07)	0.03 (−0.03–0.08)	Very-most likely trivial
**a)**	**Expertise**					
	Novice	49.9 (47.4–52.3)	-	.03 (−0.05–0.11)	−0.21 (−0.39–−0.04) *	Very likely trivial
	Non-expert	52.6 (51.2–54.1)	0.01 (−0.21–0.23)	0.00 (−0.09–0.09)	0.01 (−0.09–0.10)	Unclear
	Expert	53.8 (51.7–56.0) †	−0.04 (−0.11–0.03)	0.02 (−0.08–0.11)	0.03 (−0.05–0.11)	Likely-very likely trivial
**b)**	**ASRM motivation**					
	Autonomous	52.6 (51.4–53.7)	−0.01 (−0.05–0.03)	0.05 (−0.01–0.10)	0.06 (0.00–0.11) *	Very-most likely trivial
	Controlled	51.5 (48.9–54.1)	-	0.47 (−0.30–1.25)	0.50 (−0.30–1.29)	Unclear
**Extrinsic motivation**		42.7 (41.6–43.8)	0.12 (0.07–0.18) **	0.10 (0.03–0.17) *	0.14 (0.08–0.21) **	Most likely trivial
**a)**	**Expertise**					
	Novice	40.4 (37.9–42.9)	-	0.00 (−0.11–0.11)	−0.20 (−0.48–0.07)	Unclear-likely trivial
	Non-expert	43.8 (42.3–45.2) †	0.15 (−0.05–0.36)	0.12 (−0.02–0.27)	0.17 (0.02–0.31) *	Very-most likely trivial
	Expert	42.1 (39.9–44.3)	0.10 (0.07–0.12) **	0.08 (0.05–0.12) **	0.11 (0.06–0.15) **	Most likely trivial
**b)**	**ASRM motivation**					
	Autonomous	42.4 (41.2–43.5)	0.03 (−0.02–0.09)	0.01 (−0.05–0.07)	0.03 (−0.03–0.09)	Very likely trivial
	Controlled	44.3 (41.7–46.9)	-	−0.23 (−0.52–0.07)	−0.19 (−0.48–0.11)	Likely trivial
**Amotivation**		19.4 (18.7–20.2)	1.00 (0.00–1.01) *	1.00 (0.00–1.01) *	1.00 (0.00–1.01) *	Likely-very likely
**a)**	**Expertise**					
	Novice	19.1 (17.4–20.9)	-	−1.00 (−1.01–0.00)	−1.01 (−1.02–1.00)	Likely
	Non-expert	19.2 (18.2–20.2)	−1.01 (−1.02–1.01)	−1.00 (−1.01–1.00)	−1.00 (−1.01–1.00)	Unclear
	Expert	20.2 (18.7–22.0)	0.00 (−1.00–1.00)	0.00 (−1.01–1.01)	0.00 (−1.00–1.01)	Most likely trivial
**b)**	**ASRM motivation**					
	Autonomous	19.1 (16.2–19.9)	−1.00 (−1.00–0.00)	−1.00 (−1.01–0.00)	−1.00 (−1.00–0.00)	Unclear
	Controlled	21.1 (19.1–23.2)	-	1.01 (−1.01–1.04)	1.02 (−1.01–1.04)	Unclear

B = modelled slope for every 1% increase in ASRM compliance. Significance * *p* < 0.05, ** *p* < 0.001 compared to: * same cohort at baseline, † novice, ‡ non-expert, § autonomous.
